# Decreasing recurrence and increasing survival rates in patients of ethmoid or sphenoid intestinal-type adenocarcinomas: Systematic review and meta-analysis with 1126 cases

**DOI:** 10.1097/MD.0000000000027341

**Published:** 2021-10-08

**Authors:** Ethan I. Huang, Ang Lu, Yao-Te Tsai, Ting-Chung Wang, Huei-Chieh Chuang, Wen-Cheng Chen, Ping-Tsung Chen

**Affiliations:** a Department of Otolaryngology, Chang Gung Memorial Hospital, Chiayi, Taiwan; b School of Medicine, Chang Gung University, Taoyuan, Taiwan; c Department of Neurosurgery, Chang Gung Memorial Hospital, Chiayi, Taiwan; d Department of Pathology, Chang Gung Memorial Hospital, Chiayi, Taiwan; e Department of Radiation Oncology, Chang Gung Memorial Hospital, Chiayi, Taiwan; f Department of Hematology and Oncology, Chang Gung Memorial Hospital, Chiayi, Taiwan.

**Keywords:** craniectomy, craniofacial resection, malignancy, neoplasm, rhinotomy, skull base

## Abstract

**Background:**

Ethmoid or sphenoid intestinal-type adenocarcinomas (ITACs) form a distinct subtype of sinonasal adenocarcinomas that occur less than 1 case/100,000/yr. They have obvious exposure relationship to hardwood or leather dusts, infrequent metastasis, but a relatively high local-recurrence rate. They locate at sinuses close to vital structures listed as high-risk areas in surgeries. Even in expert hands, a craniofacial resection is associated with non-negligible mortality and morbidity. Management of these tumors, first or recurrent, needs to weigh these consequences versus the survival, regional-recurrence, and distant-recurrence rates. Due to the rareness of ethmoid or sphenoid ITACs, accurate overall survival and local- or regional-recurrence rates across diverse treatments are unclear. The aim of this study is to report the overall statistics of this cancer and the relationship between enrollment year versus age, recurrence, and survival.

**Methods:**

Systemic review and meta-analysis with 1126 cases across various treatments in the literature.

**Results:**

Here, we show that patients of ethmoid or sphenoid ITACs had overall local-, regional-, and distant-recurrence rates of 32.2%, 2.2%, and 10.3%, respectively, with a 5-year overall survival rate of 66.2%. The results present a significant correlation between age, local-recurrent rate, or overall survival rate versus enrollment year.

**Conclusion:**

This suggests that recent patients of ethmoid or sphenoid ITACs may present at an older mean age, have a lower local-recurrence rate, and have a better 5-year survival rate than before. There was a shifting trend of treating ethmoid ITACs from external approach to endoscopic resection. Clinicians may want to weigh mortality and morbidity rates of external surgeries and these data to share or decide a solution.

## 1 Introduction

Sinonasal adenocarcinomas are rare, with an annual incidence of <1 in 100,000 people per year.^[1,2]^ Sinonasal adenocarcinomas are classified by the World Health Organization into 2 histologic forms: intestinal-type adenocarcinomas (ITACs) and non-ITACs.^[3]^ ITACs can be divided^[4,5]^ into well-differentiated (papillary, tubular, and papillary-tubular type), moderately differentiated (papillary-mucinous and papillary-tubular–mucinous type), and poorly differentiated (mucinous, alveolar goblet cell, and signet-ring type).

ITACs form a distinct subtype. They have special genetic characteristics (e.g., see Llorente et al^[6]^ for a review). ITACs originate mostly in the ethmoid sinus^[7]^ (or arise from the olfactory niche^[8]^), whereas non-ITACs occur in the other paranasal sinuses. Only ethmoid ITACs have an obvious relationship with the exposure to hardwood or leather dusts.^[7]^ This exposure relationship did not present in maxillary adenocarcinomas.^[7]^ ITACs seem to show less first or recurrent regional and distant metastasis than other head-and-neck cancers.^[2,4,9–11]^ So, a routine neck dissection is not suggested for patients with a clinically negative neck.^[12]^ Ethmoid ITACs seem to show long-term mortality lower than non-ITAC (e.g., see Meccariello et al^[13]^ or Fig. 1 of Cantu et al^[7]^). When relapse occurred, the first, second local recurrence, or even regional metastasis might not affect overall survival (e.g., see Table 3 of Camp et al^[11]^).

Researchers have not reached a consensus on the approach of surgical resection for ITACs. Based on ITACs are locally aggressive tumors that easily infiltrate the underlying bone,^[14]^ some advocated a radical resection (e.g., see Cantu et al^[7]^ proposing at least a total ethmoidectomy). Researchers advocating radical resection reported that the previous treatment before the radical resection was an adverse prognostic factor. They believed the first treatment is often the only treatment (e.g., see Cantu et al^[7]^). When tumors involve the cribriform plate, external radical resections such as anterior craniofacial resection^[15]^ was said the established gold standard.^[16]^

But some studies reported patients survived long-term periods with a local disease (e.g., see Barnes^[4]^). With similar tumor stage, pathological differentiation, and adjuvant therapies, Grosjean et al^[17]^ showed significant lower morbidity by endoscopic than transfacial surgeries, yet no-different survival and local control. When properly planned and performed by an experienced surgeon, endoscopic or endoscope-assisted surgery is a valid and efficient treatment with low morbidity in most cases of ITAC.^[9–11,13,17–20]^ With the advancement in endoscopic equipment and surgeon skill, endoscopic surgery can be a practical and reliable choice for larger tumors.^[21,22]^

Ethmoid or sphenoid ITACs have a high local recurrent rate. Some series reported a high local recurrence rate being equal to or higher than 50%.^[4,7,23]^ Ethmoid, sphenoethmoid, or sphenoid cells are close to vital structures and are listed as high-risk areas in surgeries.^[24,25]^ There are long-term, intermediate, and immediate complications, including (ordered by higher incidence): serous otitis media, cerebrospinal-fluid leak, bone necrosis or fistula, epilepsy, diplopia, confusion, cellulitis, encephalitis, decreased vision, etc.^[16]^ Data from an international collaborative study on anterior craniofacial resection showed that, even in expert hands, the procedure is associated with non-negligible mortality (4.7%) and morbidity (36.3%).^[26]^ Clinicians weigh these mortality and morbidity rates versus the survival, regional-recurrence, and distant-recurrence rates of ITACs to share or decide a solution. However, due to the rareness of ethmoid or sphenoid ITACs, accurate overall survival and local- or regional-recurrence rates across diverse treatments are unclear. The aim of this study is to report the overall statistics of this cancer and the relationship between enrollment year versus age, recurrence, and survival.

## 2 Materials and method

The materials were from English literature, following the updated guideline of Preferred Reporting Items for Systematic Reviews and Meta-Analyses,^[27]^ we used multiple quantitative analyses to integrate and describe the available studies in literature and form overall statistics on survival and metastasis rates across various reported treatments. We sought possible trends by plotting the distribution and testing the correlation between various outcomes and their years, to predict whether an outcome today could be better than or no-different from those overall ones. Other worth-noting overall statistics of ethmoid or sphenoid ITACs were also presented, including age, sex, histology, and the tumor stage at presentation.

We tried to search and get a complete database of ethmoid or sphenoid intestinal-type adenocarcinoma. The often-used databases such as PubMed have a concern of lacking the function of full-text searching, except for Google Scholar. Google Scholar probably having the most records and was ranked as the most comprehensive academic search engine,^[28]^ bringing a significant amount of sources that were not previously visible.^[29]^ The search in Google Scholar in October 2020 with intestinal-type adenocarcinoma resulted in 30,500 items, excluding patents and citations. These results included the tumor subtype at other sites such as lung or gallbladder. To achieve the goal of the search strategy, the executed search version used the phrase of “nose OR nasal OR sinus OR nasosinal OR ethmoid OR sphenoid intestinal-type adenocarcinoma.” This pulled out 3720 results.

One by one in these pulled results, Huang screened an original research article if it detailed information of the patients of ITAC or can be calculated from the figures or tables, including case number, mean age, sex, treatments, overall survival, and disease recurrence. Thus, studies of sinonasal adenocarcinomas without detailed ITAC information^[18,30]^ were not included. We include an individual from an article if the above data can be obtained from the text or tables. We selected potentially relevant articles through the title and abstract for full review and also through the references cited in the reviewed articles. Exclusion criteria for an individual or article were: no tumor extension in ethmoid or sphenoid (e.g., the 3 cases in Lee et al^[31]^). Duplicated cases of a same team or from a same institute.^[10,20,32,33]^ Articles and individuals met the above criteria were classified by the year of their enrollments to investigate the evolution of outcome. Two enrollment years in one article yielded 2 records, such as external and endoscopic surgeries by a same team.

Each record included the case numbers of age, sex, histology, tumor stage at presentation, treatments, survivals, and tumor recurrences. We studied overall survival because this fundamental statistic was mostly reported. The number of the survived cases was recorded from the reported text or estimates from the figures if not reported in text. To prevent misleading results caused by different tumor stage at presentation along the years across treatment, we classified the tumor stage at presentation into advanced (T3 and T4) and non-advanced. We investigated the possible trends along the years by use of the middle of the start and end years of enrollment in each record. We plotted the distribution and examined the Spearman rank correlation between the middle of the enrollment years and each variable: age, advanced stage, local-recurrence rate, regional-recurrence rate, distant-recurrence rate, and 5-year overall survival, respectively. The statistical significance was all tested as α = 0.05.

We performed the statistical tests in MATLAB 9.4.0.813654 (MathWorks, Natick, MA).

### 2.1 Ethical statement

An ethical approval was waived because the study is a systematic review with metastasis with articles in the literature.

## 3 Results

**Figure 1:**
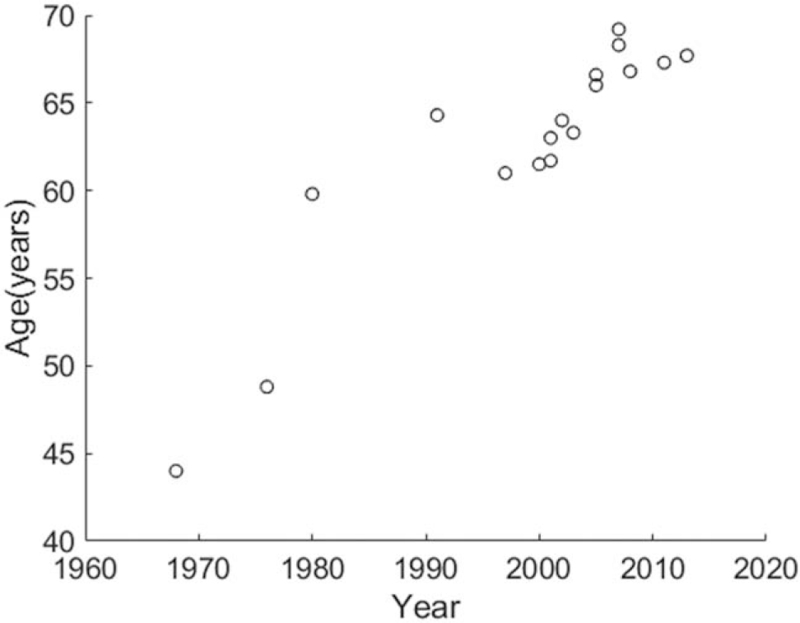
Distribution and relationship between age and the middle of enrollment years. There was a relationship that age was increasing along the years, Spearman rank correlation = 0.907, *P* < .001.

**Figure 2:**
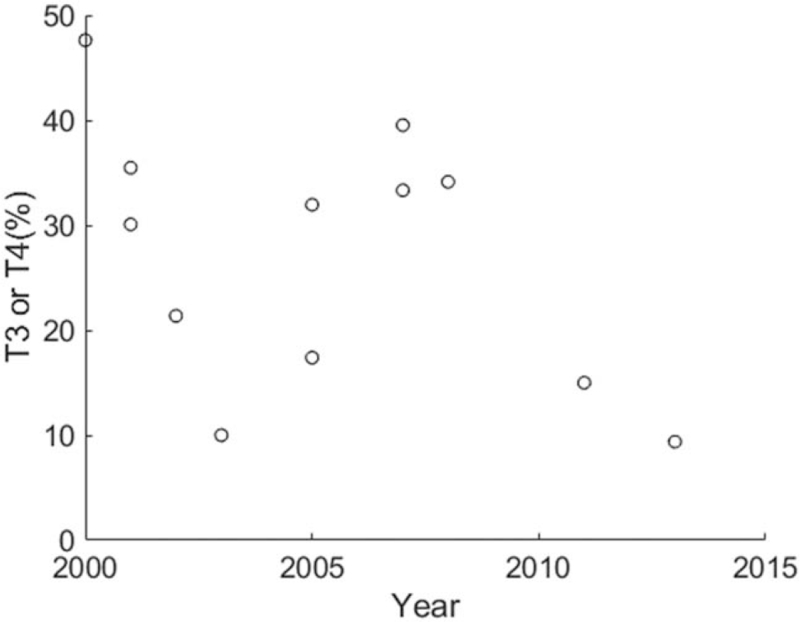
Distribution and relationship between advanced tumor stage and the middle of enrollment years. At presentation, advanced tumor stage was not related to the enrollment year, Spearman rank correlation = –0.401, *P* = .197.

Eighteen records formed from 16 articles were extracted from the 3720 pulled-out results (Table 1). They reported 1126 patients with ethmoid or sphenoid ITACs. Among these 1126 cases, 5.3% were women, and 94.7% were men. The overall mean age at presentation was 64.7 years. A Spearman rank correlation for the data revealed a relationship that age was increasing along the years, *r* = 0.907, *P* < .001 (Fig. 1). Twelve records in 9 studies^[9,11,12,17,19,22,23,34,35]^ provided tumor stage. Figure 2 displays the distribution of the 12 records of T3 or T4 stage versus enrollment year. The tumor stage was not related to the enrollment year, Spearman rank correlation = –0.401, *P* = .197.

**Table 1:** The records in this study.

Ref	Author	Publish year	Middle year	Institute	Case number
^[4]^	Barnes, L.	1986	1968	University of Pittsburgh Medical Center, USA	2
^[7]^	Gantu, G.	2011	1997	Instituto Nazionale dei Tumori, Italy	153
^[9]^	Nicolai, P.	2016	2005	University of Brescia, Italy	169
^[19]^	Antognoni, P.	2015	2007	Ospedale di Circolo e Fondazione Macchi, Italy	30
^[17]^	Grosjean, R.	2015	2001	de Nancy-Hospital Central, France	31
^[17]^	Grosjean, R.	2015	2007	de Nancy-Hospital Central, France	43
^[36]^	Alessi, D.	1988	1976	Jonsson Comprehensive Cancer Center, USA	4
^[37]^	Lopez. J.	1990	1980	Universidad del Pais Vasco, Spain	5
^[21]^	Jardeleza, C.	2009	2003	University of Adelaide, Australia	10
^[12]^	Donhuijsen, K.	2016	2002	Academic Hospital, Germany	117
^[12]^	Donhuijsen, K.	2016	2008	Academic Hospital, Germany	252
^[23]^	Franchi, A.	2011	NA	University of Florence Medical School, Italy	62
^[34]^	Breheret, R.	2011	2000	Angers University Hospital, France	42
^[38]^	Orvidas, L.	2005	1991	Mayo Clinic, USA	8
^[11]^	Camp, S.	2016	2001	University Hospitals Leuven, Belgium	123
^[22]^	Mortuaire, G.	2016	2005	Universite de Lille 2, France	23
^[22]^	Mortuaire, G.	2016	2011	Universite de Lille 2, France	20
^[35]^	Maffeis, V	2020	2013	the University of Padova, Italy	32

Ref: the reference number in this study. Middle year = the middle of the start and end years of enrollment in each record, NA = not available. It is defined as the research time (year in the figures) to test the relationship with the other variable (e.g., local recurrence or 5-year survival). We listed the institutes to exclude duplicated cases that affect the accuracy.

**Figure 3:**
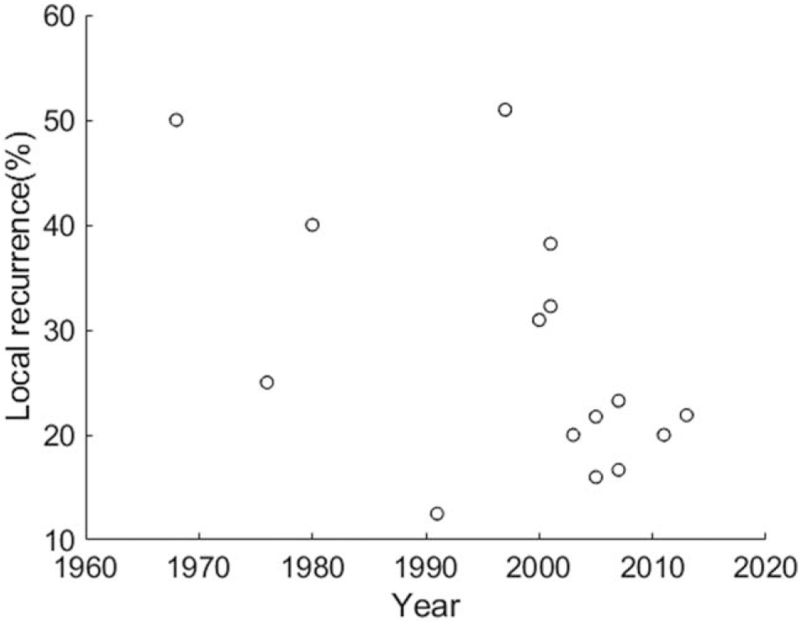
Distribution and relationship between local-recurrence rate and the middle of enrollment years. Local-recurrence rates were negatively related to years, Spearman rank correlation = –0.529, *P* = .043.

**Figure 4:**
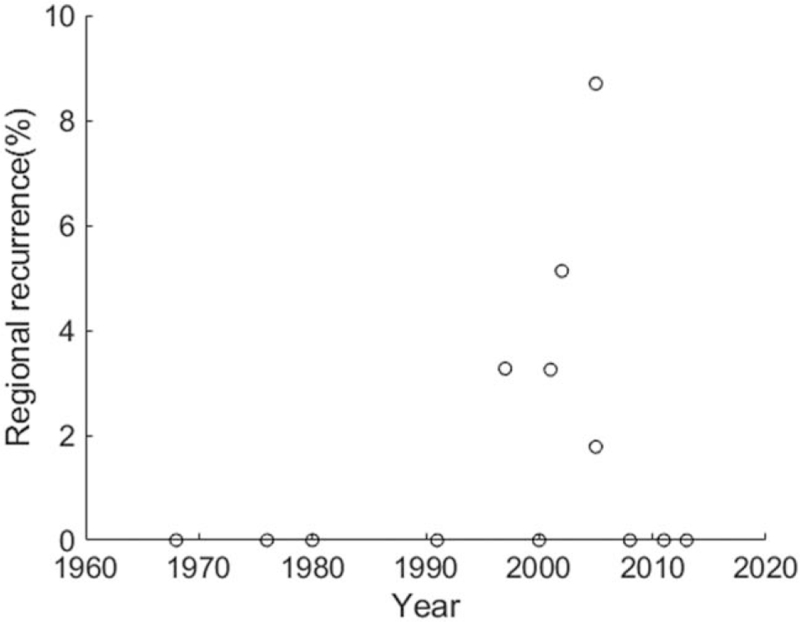
Distribution and relationship between regional-recurrence rate and the middle of enrollment years. Regional-recurrence rate was not related to the enrollment year, Spearman rank correlation = 0.169, *P* = .58.

**Figure 5:**
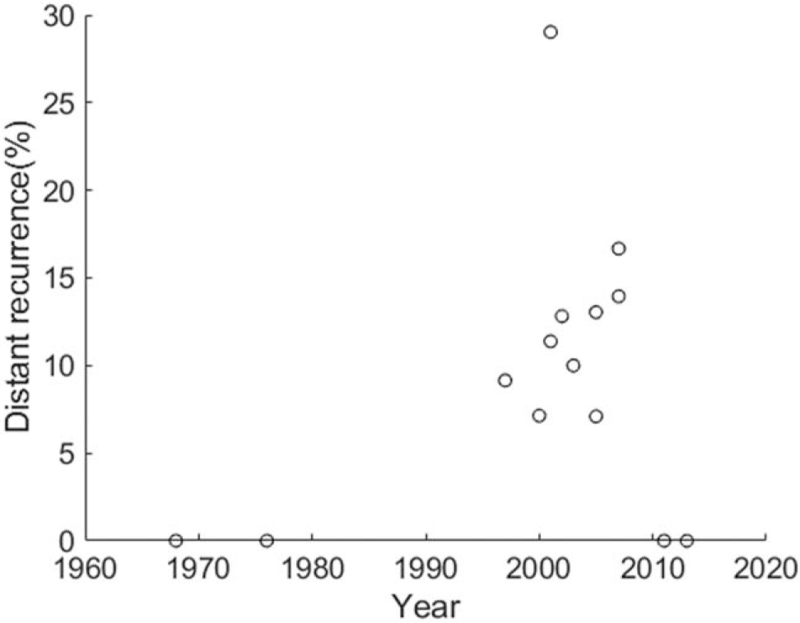
Distribution and relationship between distant-recurrence rate and the middle of enrollment years. Distant-recurrence rate was not related to the enrollment year, Spearman rank correlation = 0.145, *P* = .621.

**Figure 6:**
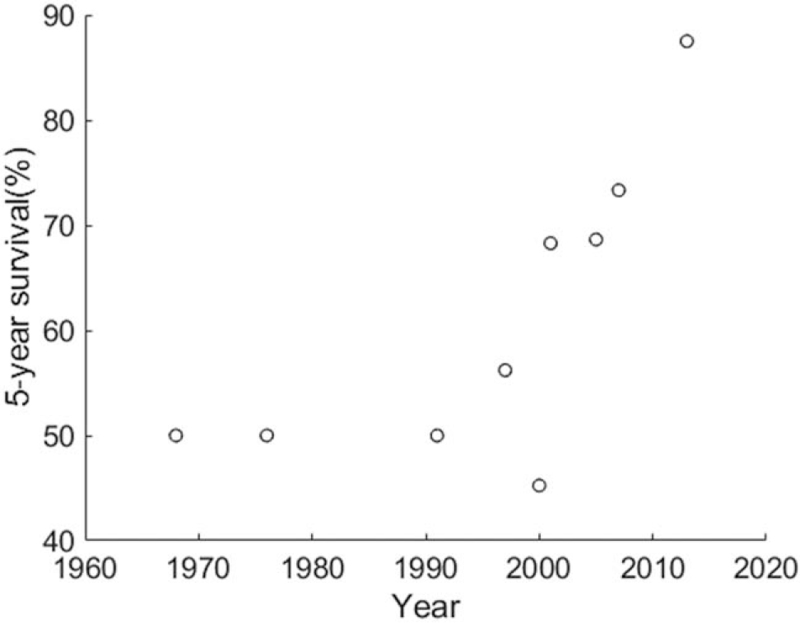
Distribution and relationship between 5-year overall survival rate and the middle of enrollment years. There was a relationship that 5-year overall survival rate was increasing along the years, Spearman rank correlation = 0.814, *P* = .011.

The overall local-recurrence rate was 32.2%, polled from 757 patients.^[4,7,9–11,17,19,21–23,34–38]^ A Spearman rank correlation for the data revealed the relationship that local-recurrence rate was decreasing along the years, *r* = –0.529, *P* = .043 (Fig. 3). The overall regional-recurrence rate was 2.2%, polled from 1022 patients in 13 records.^[4,7,9–12,22,23,34–38]^ In these 13 records, 8 reported 0% regional metastasis after treatments. A Spearman rank correlation for the data showed that regional-recurrence rate was not related to the enrollment years, *r* = 0.169, *P* = .58 (Fig. 4). The overall distant-recurrence rate was 10.3%, polled from 861 patients in 14 records.^[4,7,9–11,17,19,21–23,34–36]^ In these 14 records, 4 reported 0% distant metastasis after treatments. A Spearman rank correlation for the data indicated that regional-recurrence rate was not related to the enrollment years, *r* = 0.145, *P* = .621 (Fig. 5). The overall 5-year overall survival rate was 66.2%, polled from 606 patients.^[4,7,9,11,19,34–36,38]^ A Spearman rank correlation for the data showed the relationship that 5-year overall survival rate was increasing along the years, *r* = 0.814, *P* = .011 (Fig. 6). Table 2 lists essential overall statistics and the sources where they were extracted. A complete database can be found in the Zenodo repository (DOI:10.5281/zenodo.4103823 or https://zenodo.org/record/4103823).

**Table 2:** Overall statistics of patients with ethmoid or sphenoid intestinal-type adenocarcinomas.

Ref		%	Total (mean)	Cases
^[4,7,9,11,12,17,19,21–23,34–38]^	Age		(64.7)	1126
^[4,7,9,11,12,17,19,21–23,34–38]^	Male	94.7	1066	1126
^[4,7,9,11,12,17,19,21–23,34–38]^	Female	5.3	60	1126
^[11,17,19,21–23,34,35]^	Mucinous	32	133	416
^[9,12,19,21,23,35,36,38]^	Well diff.	14.4	62	432
^[9,12,19,21,23,35,36,38]^	Moderately diff.	57.6	249	432
^[9,12,19,21–23,35,36,38]^	Poorly diff.	23.6	112	475
^[9,11,12,17,22,34]^	Initial N0	98.9	811	820
^[9,11,12,17,22,34]^	Initial N+	1.1	9	820
^[11,12,22,34]^	Initial M1	2.2	10	460
^[4,7,9,10,17,19,21–23,36,38]^	3-year survival	72.8	404	555
^[4,7,9,11,19,22,34–36,38]^	5-year survival	66.2	401	606
^[4,7,11,38]^	10-year survival	49	140	286
^[4,7,9–11,17,19,21–23,34–38]^	Local recur.	32.2	244	757
^[4,7,9–12,22,23,34–38]^	Regional recur.	2.2	22	1022
^[4,7,9–11,17,19,21–23,34–36]^	Distant recur.	10.3	89	861

Ref: reference number of this study. diff. = differentiation, M1 = positive distant metastasis, N0 = no regional nodal metastasis, N+ = with regional nodal metastasis, recur. = recurrence.

## 4 Discussion

The results of overall statistics show that patients with ethmoid or sphenoid ITACs presented at a mean age of 64.7 years and were about 95% men. Ninety-eight of the patients presented with no regional nodal metastasis across various treatments and enrollment years. The overall local-, regional-, and distant-recurrence rates were 32.2%, 2.2%, and 10.3%, respectively, with a 5-year overall survival rate of 66.2%. These overall statistics picture an approximate behavior of the rare ethmoid or sphenoid ITACs across different treatments. The positive relationship between age at presentation and the enrollment year reflects not necessarily the behavior change of the tumor along the years. Other developing factors such as life span or advancement of technique or experience to treat older patients can also be the influencing variables. The correlation results showed the relationships of decreasing local-recurrence rate and improving 5-year overall survival rate along the years. Our results suggest a general outcome today would have a local-recurrence rate lower than 32.2%, relatively rare regional- and distant-recurrence, and a 5-year overall survival rate better than 66.2% with the advancement of techniques, if not having the ceiling effect (i.e., age reaches or is limited by the life span).

Barnes^[4]^ conducted another meta-analysis in 1986 showing data pooled about 35 years ago. Among 213 cases of ITAC, the local-, regional-, and distant-recurrence rates were 53%, 8%, and 13%. The survival rate was 40%. These confirm the lower local-recurrence rate and better survival rate today, the trends found in the present study. But the regional- and distant-recurrence rates were seemingly higher than we reported. The non-significance could be due to the small size of number (because of the rareness of the disease and metastases) or a floor effect (e.g., in Fig. 4, most records showed 0% regional metastasis).

Ethmoid or sphenoid ITACs are immunohistochemically and histologically similar to colorectal adenocarcinomas, which have dominant lymphogenic metastatic spreading. However, metastases in ITACs are more often hematogenic than lymphogenic.^[12]^ Regional metastases in ITACs happen far less frequently than they do in colorectal adenocarcinomas, ranging from 0% to 12%.^[4,10,12,23,34,39,40]^ The low rate of lymphatic or regional metastasis of ITACs was proposed to be due to in part of the particular anatomy of the ethmoid or sphenoid cells surrounded only few lymphatic vessels.^[12,41]^ This needs future studies to discover the underlying mechanism of relative rareness of regional metastasis of ethmoid or sphenoid ITACs.

We did not compare the outcomes between external and endoscopic or endoscope assisted surgeries. It is difficult, if not impossible under ethic regulations, to conduct this comparison. Without an experimental design, researchers might perform craniectomies for larger tumors and do endoscopic surgeries for small ones. Researchers could do endoscopic surgeries as technique advanced and enough experience accumulated. The comparison usually underwent in separated 2 time periods (e.g., see Ref.^[17,32]^). The difficulties include the controls of double blindness, the disease severity, and the accumulated ability and advancement of technique. There was a shifting trend of treating ethmoid ITACs from an external approach to endoscopic resection without or with endoscopic craniectomy. Lesions with no critical relationship with the orbit and anterior skull base were considered appropriate to endoscopic resection.^[9]^ Very mild or limited infiltration of the anterior skull base, focal infiltration of the dura, with no or limited intradural extension, were considered indications for endoscopic resection with endoscopic craniectomy.^[9]^ The main advantages of endoscopic over external approaches are the possibility to define the area of insertion and the tumor extension, the avoidance of brain retraction when an intradural resection is needed, the absence of external scars, and limited morbidity.^[9]^ No or very few postoperative deaths were observed in large-scale studies of endoscopic surgery.^[11,18]^

Donhuijsen et al^[12]^ proposed the most frequent cause of death being aggressive local growth of the ethmoid ITAC infiltrating the brain followed by multiple metastases at various locations. Patients getting a local recurrence could be treated with curative intent endoscopic approach.^[9–11,22]^ The endoscopic approach could be applied even in majority patients with a second or third recurrence.^[11]^ Reporting a single-institute comparison between endoscopic and external groups, Grosjean et al^[17]^ showed an at-least 30% successful rate of salvage endoscopic operation for a local recurrence in their endoscopic group. All patients presenting a local recurrence died in the transfacial group, although the 3-year local control rates were not statistically different between the 2 groups. It requires caution when reading these reports, considering the limitation of the design and the rareness of the disease. Our results show that ethmoid ITACs have a relatively high (32.2%) overall local-recurrence rate across different treatments along the years. We suggest future studies to discuss how a local recurrence can be better managed.

## 5 Conclusions

Using multiple meta-analyses with 1126 cases across treatments, our results show that patients of ethmoid or sphenoid ITACs presented at an overall mean age of 64.7 years and were about 95% men. Across various treatments, the overall local-, regional-, and distant-recurrence rates were 32.2%, 2.2%, and 10.3%, respectively, with a 5-year overall survival rate of 66.2%. The results also present a significant correlation between age, local-recurrent rate, and overall survival rate versus enrollment year. This suggests that recent patients of ethmoid or sphenoid ITACs may present at an older mean age, have a lower local-recurrence rate, and have a better 5-year survival rate. There was a shifting trend of treating ethmoid ITACs from an external approach to endoscopic resection. Clinicians may want to weigh mortality and morbidity rates of external surgeries and these data to share or decide a solution.

## Author contributions

Ethan I. Huang conceptualized the study, collected data, ran analysis, and wrote the first draft of the manuscript. Ang Lu and Yao-Te Tsai reviewed the data, provided essential comments on the manuscript, and revised the manuscript. Ting-Chung Wang commended on neurosurgeries and revised the manuscript. Huei-Chieh Chuang commended on pathology and revised the manuscript. Wen-Cheng Chen and Ping-Tsung Chen commended on the data and revised the manuscript.

**Conceptualization:** Ethan I. Huang.

**Data curation:** Ang Lu, Yao-Te Tsai.

**Formal analysis:** Ethan I. Huang.

**Methodology:** Ethan I. Huang.

**Resources:** Yao-Te Tsai, Ting-Chung Wang, Huei-Chieh Chuang, Wen-Cheng Chen, Ping-Tsung Chen.

**Software:** Ethan I. Huang.

**Validation:** Yao-Te Tsai, Huei-Chieh Chuang.

**Visualization:** Ang Lu, Huei-Chieh Chuang.

**Writing – original draft:** Ethan I. Huang.

**Writing – review & editing:** Ang Lu, Yao-Te Tsai, Ting-Chung Wang, Huei-Chieh Chuang, Wen-Cheng Chen, Ping-Tsung Chen.
